# Modeling Consonant-Vowel Coarticulation for Articulatory Speech Synthesis

**DOI:** 10.1371/journal.pone.0060603

**Published:** 2013-04-16

**Authors:** Peter Birkholz

**Affiliations:** 1 Department of Phoniatrics, Pedaudiology, and Communication Disorders, University Hospital Aachen and RWTH Aachen University, Aachen, Germany; University of Catania, Italy

## Abstract

A central challenge for articulatory speech synthesis is the simulation of realistic articulatory movements, which is critical for the generation of highly natural and intelligible speech. This includes modeling coarticulation, i.e., the context-dependent variation of the articulatory and acoustic realization of phonemes, especially of consonants. Here we propose a method to simulate the context-sensitive articulation of consonants in consonant-vowel syllables. To achieve this, the vocal tract target shape of a consonant in the context of a given vowel is derived as the weighted average of three measured and acoustically-optimized reference vocal tract shapes for that consonant in the context of the corner vowels /a/, /i/, and /u/. The weights are determined by mapping the target shape of the given context vowel into the vowel subspace spanned by the corner vowels. The model was applied for the synthesis of consonant-vowel syllables with the consonants /b/, /d/, /g/, /l/, /r/, /m/, /n/ in all combinations with the eight long German vowels. In a perception test, the mean recognition rate for the consonants in the isolated syllables was 82.4%. This demonstrates the potential of the approach for highly intelligible articulatory speech synthesis.

## Introduction

Although established speech synthesis techniques like unit-selection synthesis [1] or statistical parametric speech synthesis [2] are now able to generate natural-sounding speech, in the longer-term, articulatory speech synthesis is still widely considered as the ultimate solution to speech synthesis [3]. It is potentially much more flexible than the established techniques with respect to the simulation of specific voices, speaking styles, and emotions. In practice, however, it has proven to be difficult to generate natural-sounding speech even for a single voice and speaking style. The reason is that articulatory speech synthesis is an exceedingly complex task that requires the integration of elaborate models of the vocal tract (e.g. [4]–[12]), the vocal folds [13], the aero-acoustic simulation (e.g. [14], [15]), and articulatory control (e.g. [16]–[18]). The quality of the synthesis critically depends on the detail and realism of each individual model and their interplay.

The effective control of the model articulators is one of the major challenges in articulatory speech synthesis. A central difficulty is that speech sounds are coarticulated, i.e., the articulatory and acoustic realization of phonemes depends on the context [19]. For example, the consonant /g/ in the syllable /gu/ is articulated with a more retracted tongue body and with more rounded lips than in the syllable /gi/. In unit-selection speech synthesis, such coarticulatory variations are captured by concatenating natural speech units from a large database with realizations of each phoneme in a variety of contexts. However, for articulatory speech synthesis it is not practical to record articulatory data of phonemes in all possible phonetic contexts, because articulatory recordings and their analysis are much more intricate than acoustic recordings. Instead, articulatory speech synthesis depends on numerical models of coarticulation.

One of the earliest coarticulation models was presented by Öhman [16]. Here, the time-varying vocal tract shape (in terms of the vocal tract area function) in vowel-consonant-vowel (VCV) utterances was modeled as the superposition of a diphthongal vowel gesture spanning the two vowels and a consonant gesture. The influence of the vowel shape on an assumed ideal consonant target shape was modeled with a context-independent *coarticulation function*, which specifies the amount to which the ideal consonant shape is allowed to be distorted by the vowel shape as a function of the position along the vocal tract center line. For an alveolar stop, for example, the allowed distortion is small in the region of the tongue tip, but higher in the region of the tongue back. Öhmans work led to a number of vocal tract area function models based on the superposition principle to simulate coarticulation [20]–[22]. Birkholz et al. [12], [17] simulated coarticulation with a 3D articulatory model of the vocal tract based on a *dominance model*, which can be considered as an extension of Öhmans approach from the domain of the vocal tract area function to the articulatory domain. Here, each vocal tract parameter of a consonant is associated with a context-independent dominance value, which specifies the degree of involvement of the parameter in the formation of a closure or constriction. The less involved a parameter is, the more it is determined by the underlying vowel. Lindblom and Sussman [23] recently used a similar approach with a 2D articulatory model to analyze the cause of locus equations [24].

Fowler and Saltzman [18] consider the concept of coarticulation in the context of a task-dynamic model. In this model, the articulatory and acoustic context-sensitivity of phonemes arises primarily from the dynamic interaction of *gestures*, as defined in articulatory phonology [25]. In the utterance /da/, for example, a gesture for the realization of /d/ would be assumed to overlap in time with the vowel gesture for /a/, both competing for the control of the shared articulators tongue body and jaw. Task-dynamics involves a system to *blend* the influences of the competing gestures on the articulators based on the interaction of dynamical systems. The concept of gestures was also adopted by Kröger [26] in the context of a speech production model of German. More recently, cognitive models of speech production and perception were proposed with the aim to *learn* the context-dependent coordination of articulatory movements from acoustic training data [27]–[29].

Most of the past models aimed at the simulation of basic articulatory phenomena without the focus on perception or high-quality articulatory speech synthesis. This paper presents a novel approach to simulate consonant-vowel coarticulation effectively both at the articulatory and the acoustic level. Therefore, consonants were modeled in terms of context-sensitive articulatory targets [30]. The basic idea was to calculate the context-sensitive target of a consonant as the weighted average of reference targets of the consonant in the context of the corner vowels /a/, /i/ and /u/, i.e., by bilinear interpolation. By definition, the corner vowels represent the most extreme vowel articulations in terms of the tongue position, i.e., /a/ is produced with the tongue as low and as far back as possible, /i/ with the tongue as high and forward as possible, and /u/ with the tongue as high and as far back as possible, while all other vowels are assumed to be produced with tongue positions in between these. The corner vowels also mark the corners of the acoustic vowel space. Our assumption in this study was that the different realizations of a consonant in the context of these corner vowels accordingly reflect the extremes of its coarticulatory and acoustic variation. The reference targets for consonants, i.e., their three articulatory realizations in the context of /a/, /i/, and /u/, were obtained from real-time MRI scans and optimized with respect to their acoustic realization. The weights for averaging the reference targets were determined by mapping the actual context vowel of the consonant into the articulatory subspace spanned by the corner vowels /a/, /i/, and /u/. In this study we considered the consonants /b/, /d/, /g/, /l/, /r/, /m/ and /n/, but the extension to other consonants is straightforward. The performance of the model was evaluated in terms of the recognition rate of the consonants in CV syllables synthesized with the articulatory speech synthesizer VocalTractLab (www.vocaltractlab.de).

The vocal tract and articulations modeled in this study are based on a native speaker of German. The following section describes the data analyzed from this reference speaker. Then we describe how this data was used to create the vocal tract model and the proposed coarticulation model. Finally, we turn to the perceptual experiment using synthesized CV-syllables and discuss the results.

### Data analysis

**Figure 1:**
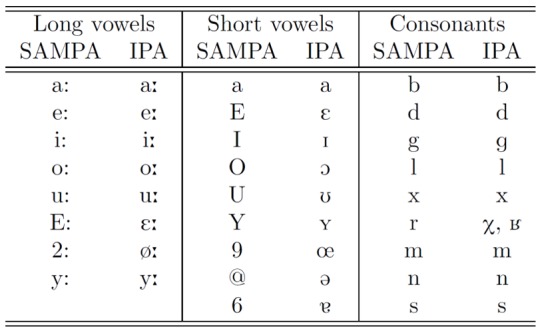
The ASCII-based speech sound symbols (SAMPA) and the corresponding symbols of the International Phonetic Alphabet (IPA) for the sounds used in this study.

All data used for modeling the vocal tract and articulation in this study were collected from the same adult native speaker of Standard German. Three types of data were analyzed: a corpus of volumetric MRI data of sustained phonemes, a corpus of real-time MRI data of CV syllables, and a corpus of high-quality audio data. Please note that due to font regulations of the journal, the ASCII-based phonetic alphabet SAMPA is used to refer to the different speech sounds [31]. Figure 1 shows the SAMPA symbols used in this paper and their corresponding symbols in the International Phonetic Alphabet (IPA).

### Volumetric MRI data and CT data

The volumetric MRI data of the vocal tract was available from a previous study [32] and consisted of sustained articulations of German vowels and consonants. This data was used to define the vocal tract model and to adjust vocal tract target shapes for vowels.

The images were acquired using a Philips Gyroscan NT scanner at the Institute for Radiology at the Virchow Clinical Center in Berlin. Each phoneme was recorded with 18 sagittal slices of 3.5 mm thickness and 512×512 pixels with a pixel size of 0.59×0.59 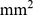. The acquisition took 21 s per phoneme. In the present study, we analyzed the data for the long vowels /a:/, /e:/, /i:/, /o:/, /u:/, /E:/, /2:/ and /y:/, and the short vowels /I/, /E/, /a/, /O/, /U/, /Y/, /9/, /@/ and /6/.

**Figure 2:**
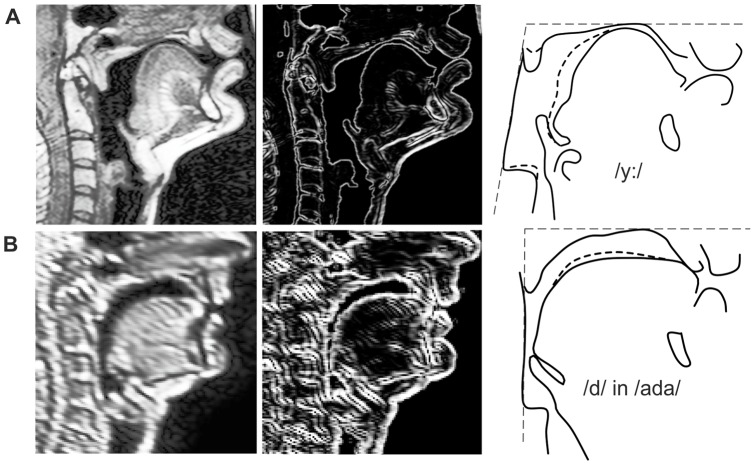
Example images from the two MRI corpora and their traced contours. A: Original midsagittal image of the vocal tract for the vowel /y:/ from the volumetric MRI corpus (left), the same image with enhanced edges (middle), and the traced contours (right). B: Same as A for an image of the real-time MRI corpus showing the consonant /d/ in /a/-context. The thick dashed lines in the traced images show the outline of the tongue side. The thin dashed lines indicate the angle of the rear pharyngeal wall with respect to the hard palate, which varies between the two corpora. The traced images were rotated for an identical orientation of the hard palate. (Figure modified from [17]).

For each of these phonemes, the vocal tract contours in the midsagittal slice were manually traced using Catmull-Rom splines. To highlight the edges in the MR images for tracing, the Sobel operator was applied to the original images. The tongue outline was not only traced in the midsagittal slice, but also in the slice about 10 mm to the left of the middle. The two outlines of the tongue were later used to reproduce the cross-sectional shape of the tongue. Finally, the mandible bone was traced in each midsagittal image as an indicator for the degree of jaw opening. Figure 2A shows as an example the midsagittal slice of the vowel /y:/, the highlighted edges, and the traced contours. Besides the sagittal contours, we measured the lateral width of the larynx and pharynx at multiple positions for phonemes with a fronted tongue position as estimates of the lateral dimensions of the vocal tract in these regions.

In addition to the vowels, we analyzed the consonants /s/ and /m/ with respect to the shape of the velum (note that /s/ is not part of the actual coarticulation study). This data was later used to define reference shapes for the maximally raised and lowered velum of the vocal tract model. Finally, plaster models of the hard palate and the mandible (including all teeth) of the speaker were scanned in a computer tomograph with a voxel size of 0.226×1×0.226 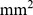. This data was used for modeling the corresponding rigid 3D structures in the vocal tract model.

### Midsagittal real-time MRI data

The real-time MRI data of the vocal tract was also available from a previous study [33] and consisted of sequences of the pseudowords /baCa/, /biCi/ and /buCu/ with different consonants C. This data was used to reproduce the context-sensitive reference shapes of the vocal tract for the consonants /b/, /d/, /g/, /l/, /r/, /m/ and /n/. Therefore, we analyzed the MRI sequences with the consonants /b/, /d/, /g/, /l/, and /x/. The velar/uvular voiceless fricative /x/ in the corpus was used as substitute to model the uvular approximant /r/, because the corpus did not contain recordings of /r/. Furthermore, the consonants /m/ and /n/ were modeled using the data for /b/ and /d/ with a lowered velum, as described further below.

The data was acquired for midsagittal sections of the vocal tract at a frame rate of 8 Hz using a Philips Gyroscan NT scanner at the Department of Radiology at the Technical University of Munich. The slice thickness was 10 mm and the resolution was 256×256 pixels with a pixel size of 1.18×1.18 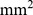. The pseudowords were produced consecutively at a normal speaking rate and repeated about ten times each. From this data, we selected the images showing the consonants /b/, /d/, /g/, /l/, and /x/ in the context of the vowels /a/, /i/ and /u/ as basis for the proposed coarticulation model. Because of the low frame rate of 8 Hz, the consonants were sampled in only some of the repeated pseudowords during the interval of the corresponding consonantal constriction. Therefore, we first visually identified the sets of frames that actually represented the context-sensitive consonantal targets, and then selected the most representative frame from each set. The most representative frame was taken to be the one with the smallest “distance” to all other images in the set (in terms of signal energy in the difference image), i.e., the most central member of the set. For example, in the 10 repetitions of /bada/ we identified three frames with a satisfactory alveolar closure for /d/. From these three frames, one frame was selected as a template for /d/ in /a/-context. In the selected frames the vocal tract contours were traced analogous to the volumetric images. Because this corpus consisted only of midsagittal slices, it contained no information about the contours of the tongue side. This information was complemented with measurements from a recent pilot study (unpublished data) where the same VCV sequences were recorded with a new real-time MRI technique [34] where two parallel sagittal slices (in the middle and 1 cm to the left) were recorded simultaneously at a frame rate of 25 Hz. Because the speaker in the pilot study was not the one modeled in this study, the contours of the tongue side must be considered as approximations. Figure 2B shows an example of a real-time MRI frame for /d/ in /a/-context and the corresponding edge and contour images.

### Normalization of head posture

**Figure 3:**
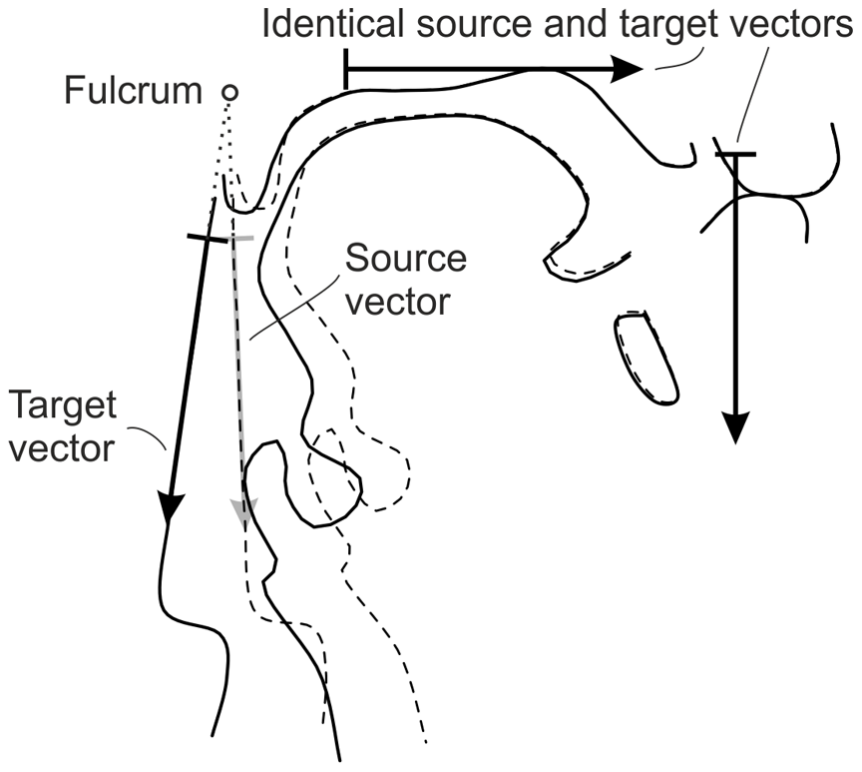
Warping of the vocal tract shape to normalize the head posture to a specific orientation of the rear pharyngeal wall based on corresponding pairs of vectors [**35**]. The source shape has a dotted outline and the target shape a solid outline. Three pairs of corresponding vectors were used to define the warping. The superior and anterior vectors were identical for the source and the target shapes, keeping these parts of the vocal tract essentially equal. The posterior vector was aligned with the rear pharyngeal wall in the source shape and rotated to the required orientation for the (normalized) target shape around a common fulcrum.

As Figure 2 illustrates, the head posture, i.e., the angle of the rear pharyngeal wall with respect to the hard palate, was not identical in both MRI corpora. Also within each corpus, the postures varied slightly. To merge both corpora for modeling, we normalized the head postures in all MRI tracings as previously described in [17]. Basically, we assumed the oral and pharyngeal parts of the vocal tract to be connected as if by a hinge joint, where different postures correspond to different hinge angles. The position of the fulcrum was determined as the point where the straight line approximations of the rear pharyngeal wall of the contour tracings from the two corpora intersected. Because all the different straight line approximations did not exactly intersect at the same position, the common fulcrum position was determined in a least-square sense [17]. After that, each MRI tracing was *warped* such that the rear pharyngeal outline was oriented at a pre-defined constant angle. Warping was implemented using the method by Beier and Neely [35] with three corresponding pairs of vectors, as illustrated in Figure 3. A horizontal vector on top of the palate and a vertical vector at the chin were identical in both the original and the warped image to preserve the vocal tract shape in these regions of the vocal tract. The third vector was aligned to the rear pharyngeal wall in the original image and rotated around the fulcrum to assume the predefined angle in the warped image.

### Acoustic recordings

The purpose of the audio recordings was to obtain a complete set of speaker-specific formant frequency targets for the German vowels and for the formant frequencies at the onset of the vowels after the consonants /b/, /d/, /g/, /l/, /r/. The consonants /m/ and /n/ were not considered in this audio corpus, because their antiresonances prevent reliable formant measurements. Because of the high noise level in the MRI scanner, these recordings were made in a separate session in a sound-proofed room for a high audio quality. To have the same postural influence on articulation as in the MRI scanner, the recordings were made with the speaker in supine position. The speech was recorded with a 44 kHz sampling rate at 16 bit quantization to a digital tape recorder using a high-quality microphone mounted on a headset.

The speaker read a number of target words (prompts), each of which was spoken in the carrier sentence “Ich habe ... gesagt. ” at a comfortable speed, pitch and loudness. The prompt list consisted of the nonsense words /CVd@/ for all combinations of the consonants /b/, /d/, /g/, /l/, /r/ and the long vowels /a:/, /e:/, /i:/, /o:/, /u:/, /E:/, /2:/, /y:/, the words /CVt@/ for all combinations of the same consonants and the short vowels /I/, /E/, /a/, /O/, /U/, /Y/, /9/, as well as the words /hOp6/, /hOk6/, and /mUt6/ with the low Schwa as final vowel. This prompt list with a total of 78 items was recorded six times (three times in each of two separate sessions) for six instances of each target word in total.

In the words /CVd@/ and /CVt@/, the first three formant frequencies 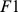, 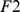, and 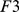 were measured at the onset of voicing of the target vowel, in the middle of the target vowel, and for the final Schwa. In addition, the voice-onset time (VOT) was determined in the words with initial plosives, measured from the closure release to the middle of the first fully established glottal period of the vowel. In the words /hOp6/, /hOk6/, and /mUt6/, only the formants of the final low Schwa /6/ were measured.

The formants were determined manually using the software Praat version 5.1.18 based on the built-in LPC formant tracker. For each word, the number of LPC poles was carefully adjusted for the best possible visual match between the peaks in the wideband spectrogram display and the superimposed LPC-based estimation. Formant frequencies at vowel onset after the initial consonants were measured at the first discernible glottal pulse after the release burst or frication phase. For the fully voiced /l/, the “onset formants” were measured in the stationary phase of the lateral. The formants of vowels were measured as mean values in the visually-determined steady state portions of the vowel. When a formant trajectory was diagonally rising or falling, the target value was taken at the midpoint of the vowel. For a U-shaped formant trajectory in the vowel portion (or the inverse), the minimum (or maximum) frequency was taken as target. About 1% of the formant values could not be uniquely identified or measured and were excluded from the subsequent analysis.

**Figure 4:**
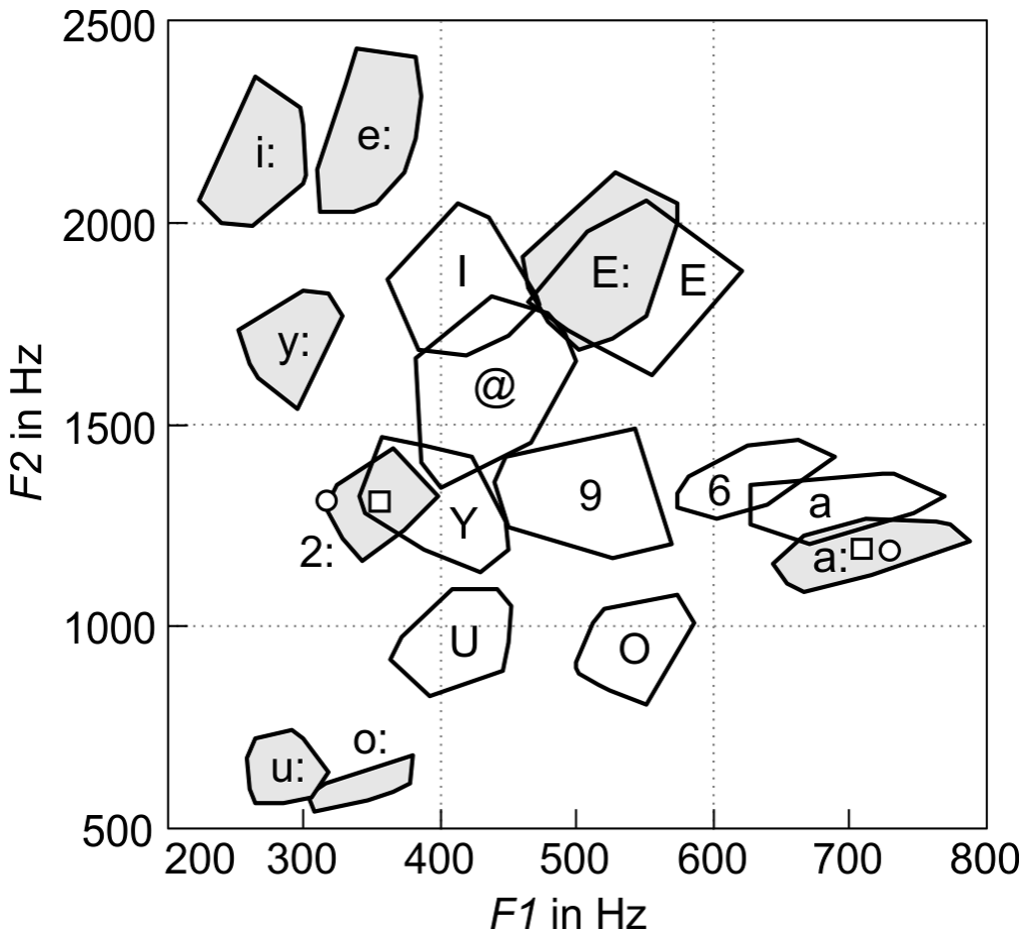
Convex hulls of the samples measured for the individual German vowels spoken in different contexts in the 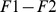 formant plane. The gray and white regions surround the long and short vowels, respectively. For each vowel, the mean formant frequencies of the analyzed samples were taken as the underlying acoustic target for the vowel according to the undershoot model [42]. Exceptions were the vowels /2:/ and /a:/, for which the mean formant values (white squares) did not represent perceptually high-quality targets. Instead, the acoustic targets for these vowels were obtained from additionally recorded sustained /2:/ and /a:/ (white circles).

**Figure 5:**
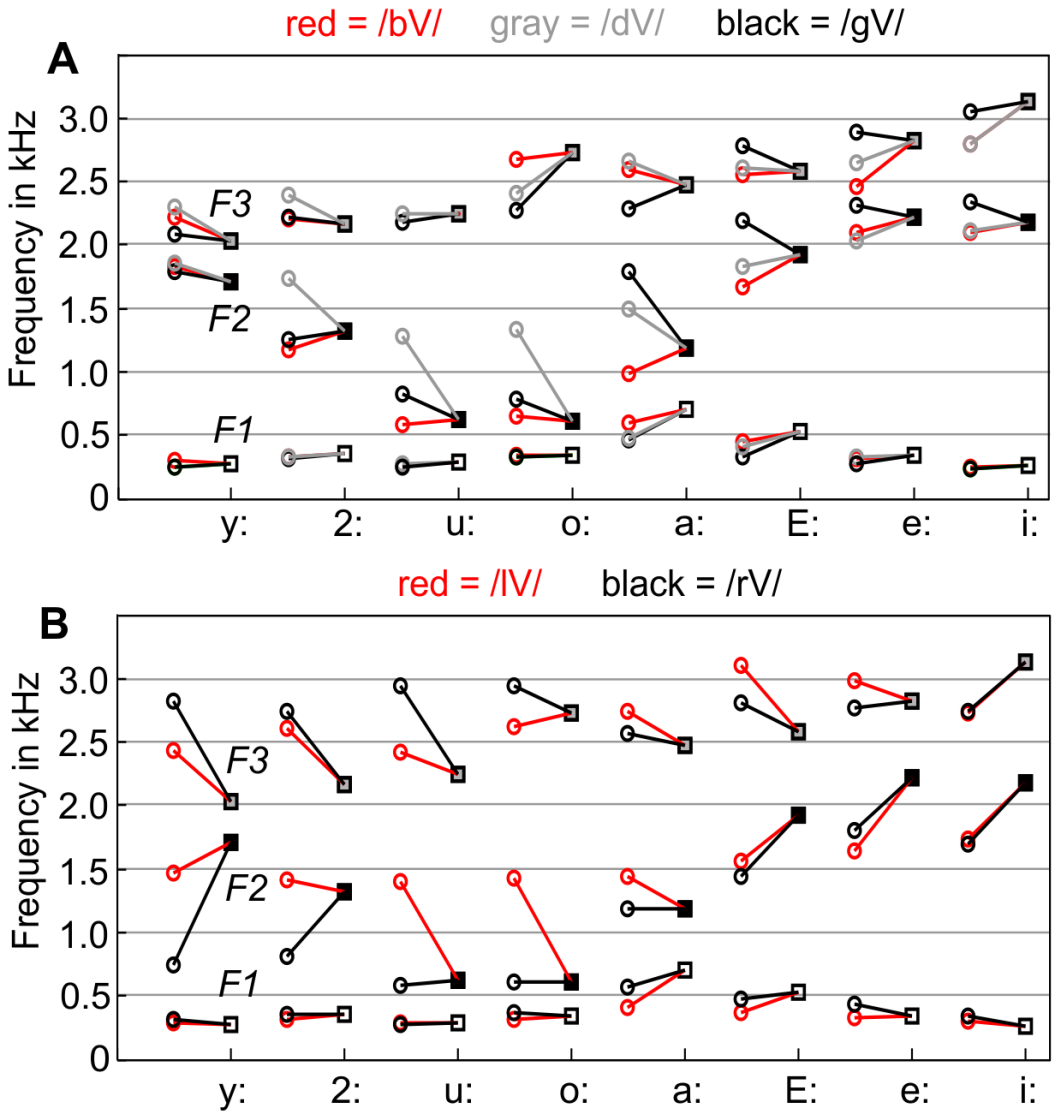
Stylized formant transitions from the consonants /b/, /d/, /g/, /l/, /r/ to the eight long German vowels of our reference speaker, based on the onset and target formant frequencies given in **Table 1**. Panel A shows the transitions for /b/, /d/, and /g/, and panel B for /l/ and /r/.

The measurement results are presented in Figure 4, 5, and Table 1. Figure 4 shows the 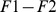 distributions for the analyzed vowels in terms of the convex hulls around the respective vowel samples. Each region was determined from 30 samples (each vowel after five different consonants with six repetitions). The regions for the long vowels are painted in gray.

**Table 1:** Results of formant frequency and VOT measurements.

	Target			Onset after /b/				Onset after /d/				Onset after /g/				Target of /l/			Onset after /r/		
	F1	F2	F3	VOT	F1	F2	F3	VOT	F1	F2	F3	VOT	F1	F2	F3	F1	F2	F3	F1	F2	F3
/a:/	716	1184	2814	14.5	599	992	2591	17.5	479	1492	2663	30.5	461	1790	2290	407	1442	2738	577	1185	2570
/e:/	346	2222	2822	14	318	2096	2463	24	323	2033	2653	28	278	2313	2893	335	1641	2990	431	1798	2769
/i:/	265	2179	3127	16	244	2100	2795	27	232	2112	2798	25	232	2337	3050	296	1738	2733	348	1694	2738
/o:/	337	605	2730	18	337	648	2673	16	332	1339	2403	53.5	326	786	2269	309	1431	2628	372	616	2950
/u:/	288	628	2249	34	265	589	x	21	274	1285	2241	38.5	247	820	2180	292	1402	2421	278	577	2948
/E:/	526	1918	2582	15	453	1664	2555	21	405	1829	2612	37	324	2194	2779	375	1563	3108	472	1445	2809
/2:/	316	1311	1943	22	332	1171	2210	26	330	1734	2393	33	312	1254	2225	311	1409	2613	351	814	2742
/y:/	274	1704	2032	28	302	1827	2222	29	247	1863	2302	38.5	247	1786	2082	293	1467	2435	309	747	2820
/I/	406	1864	2551	12.5	355	1938	2518	24	352	1940	2569	35.5	324	2143	2780	311	1615	3000	424	1416	2845
/E/	532	1859	2609	13.5	473	1695	2474	19.5	409	1895	2574	39	375	2121	2752	376	1586	2742	481	1639	2767
/a/	694	1294	2395	13	604	1092	2315	18	507	1574	2631	29.5	490	1760	2216	402	1467	2833	537	1287	2730
/O/	534	929	2514	14.5	472	744	2598	19	436	1323	2591	49	425	863	2272	366	1458	2637	442	790	2707
/U/	405	951	2540	17	377	728	2692	18	351	1398	2588	57.5	353	880	2459	340	1479	2595	367	689	2755
/Y/	396	1302	2334	25	345	1226	2384	24.5	372	1603	2409	36	320	1203	2115	308	1442	2420	354	742	2665
/9/	501	1334	2338	15	411	1257	2523	19	434	1680	2478	31.5	372	1605	2195	355	1418	2694	413	840	2598
/@/	435	1614	2573																		
/6/	639	1388	2302																		

Target formant frequencies, onset formant frequencies (in Hz), and voice onset times (VOT, in ms) for the German vowels in the context of five consonants of the reference speaker. Each target formant frequency is the mean value of 30 samples, while each of the onset formants and VOTs are median values of six samples. Exceptions are the vowels /a:/ and /2:/, for which the formants were calculated from sustained phonemes (see text for details). The nasals /m/ and /n/ are omitted in this table, because their antiresonances prevent reliable formant measurements.

Figure 5 shows 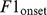, 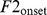, and 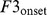 of the consonants in relation to the formant targets of the vowels. The shown formant onset values are the *median* values (because of the small sample size) of the six measured instances of each CV-combination, and the formant target values are the mean values of the 30 instances per vowel. The connecting lines represent the stylized formant transitions from the consonants to the vowels and illustrate the acoustic variability of the consonants.

Table 1 contains the numerical values for the measured formants. As in Figure 5, formant targets for vowels are mean values and formant onset values are median values. The only exceptions are the formant targets for the vowels /a:/ and /2:/, for which the mean values of the measured instances did not represent a perceptually high-quality target for the vowel according to informal listening tests using a formant synthesizer. Instead, the data for /a:/ and /2:/ in Table 1 represent the formants of additional recordings of sustained articulations of these vowels (shown by white circles in Figure 4). In general, it seems that the mean formant values of a vowel measured in different contexts does not necessarily represent an ideal target for the vowel.

## Modeling

### Vocal tract model

Using the acquired speaker data, a geometrical 3D vocal tract model was developed to represent the time-varying shape of the supraglottal airways. This 3D shape is the basis for accurate calculation of area functions of the vocal tract for the acoustic simulation. The current model is an extension of our previous vocal tract model, which was presented in detail in [12], [17], [36]. This section gives a brief overview of the model and highlights the improvements since the previous version, in particular the control of the jaw and the velum.

**Figure 6:**
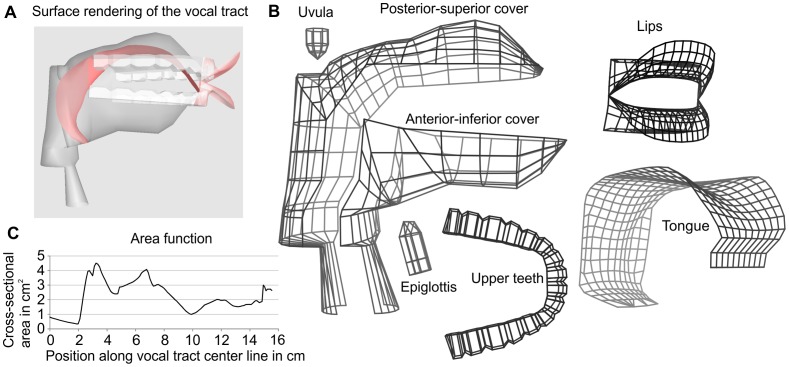
The 3D model of the vocal tract. A: Rendering of the vocal tract model for the vowel /E:/. B: Wireframe representation of the model surfaces. C: Area function of the vocal tract shape in panel A used for the acoustic simulation. The area function describes the acoustically relevant variation of the cross-sectional area of the vocal tract between the glottis (at 0 cm) and the mouth opening (here at 15.5 cm).

Generally, the vocal tract is defined in terms of a number of geometric surfaces of the articulators and vocal tract walls as illustrated in Figure 6. Their shape and position in 3D space are specified by a set of control parameters summarized in Table 2, each corresponding to one degree of freedom (DOF). The parameters were carefully defined to permit the flexibility needed to produce a large set of speech sounds, while as far as possible prohibiting anatomically impossible shapes. This was supported by geometrical constraints that, e.g., prevent interpenetrations of the articulators.

**Table 2:** Control parameters of the vocal tract model.

Name	Description	Min.	Max	Unit
*HX*	Horiz. hyoid position	0.0	1.0	
*HY*	Vert. hyoid position	−6.0	−3.5	cm
*JX*	Horiz. jaw displacement	−0.5	0.0	cm
*JA*	Jaw angle	−7.0	0.0	deg
*LP*	Lip protrusion	−1.0	1.0	
*LD*	Vert. lip distance	−2.0	4.0	cm
*VS*	Velum shape	0.0	1.0	
*VO*	Velic opening	−0.1	1.0	
*TCX*	Tongue body center X	−3.0	4.0	cm
*TCY*	Tongue body center Y	−3.0	1.0	cm
*TTX*	Tongue tip X	1.5	5.5	cm
*TTY*	Tongue tip Y	−3.0	2.5	cm
*TBX*	Tongue blade X	−3.0	4.0	cm
*TBY*	Tongue blade Y	−3.0	5.0	cm
*TRX*	Tongue root X	−4.0	2.0	cm
*TRY*	Tongue root Y	−6.0	0.0	cm
*TS1*	Tongue side elevation 1	−1.4	1.4	cm
*TS2*	Tongue side elevation 2	−1.4	1.4	cm
*TS3*	Tongue side elevation 3	−1.4	1.4	cm
*TS4*	Tongue side elevation 4	−1.4	1.4	cm
*MA1*	Min. area tongue back region	0.0	0.3	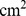
*MA2*	Min. area tongue tip region	0.0	0.3	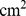
*MA3*	Min. area lip region	0.0	0.3	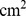

For each parameter, the value range and the unit is given. Parameters without a unit specify relative values.

The posterior-superior cover surface defines the shape of the hard palate, the velum, and the posterior wall of the pharynx and larynx. The anterior-inferior cover defines the shape of the anterior parts of the larynx and pharynx and the jaw. The remaining surfaces define the shapes of the tongue, lips, upper and lower teeth, uvula and epiglottis. The surfaces for the rigid parts of the vocal tract, i.e., the hard palate, the jaw, and the teeth, were closely adapted to the reference speakers' geometry using the CT data of the plaster models of these parts.

**Figure 7:**
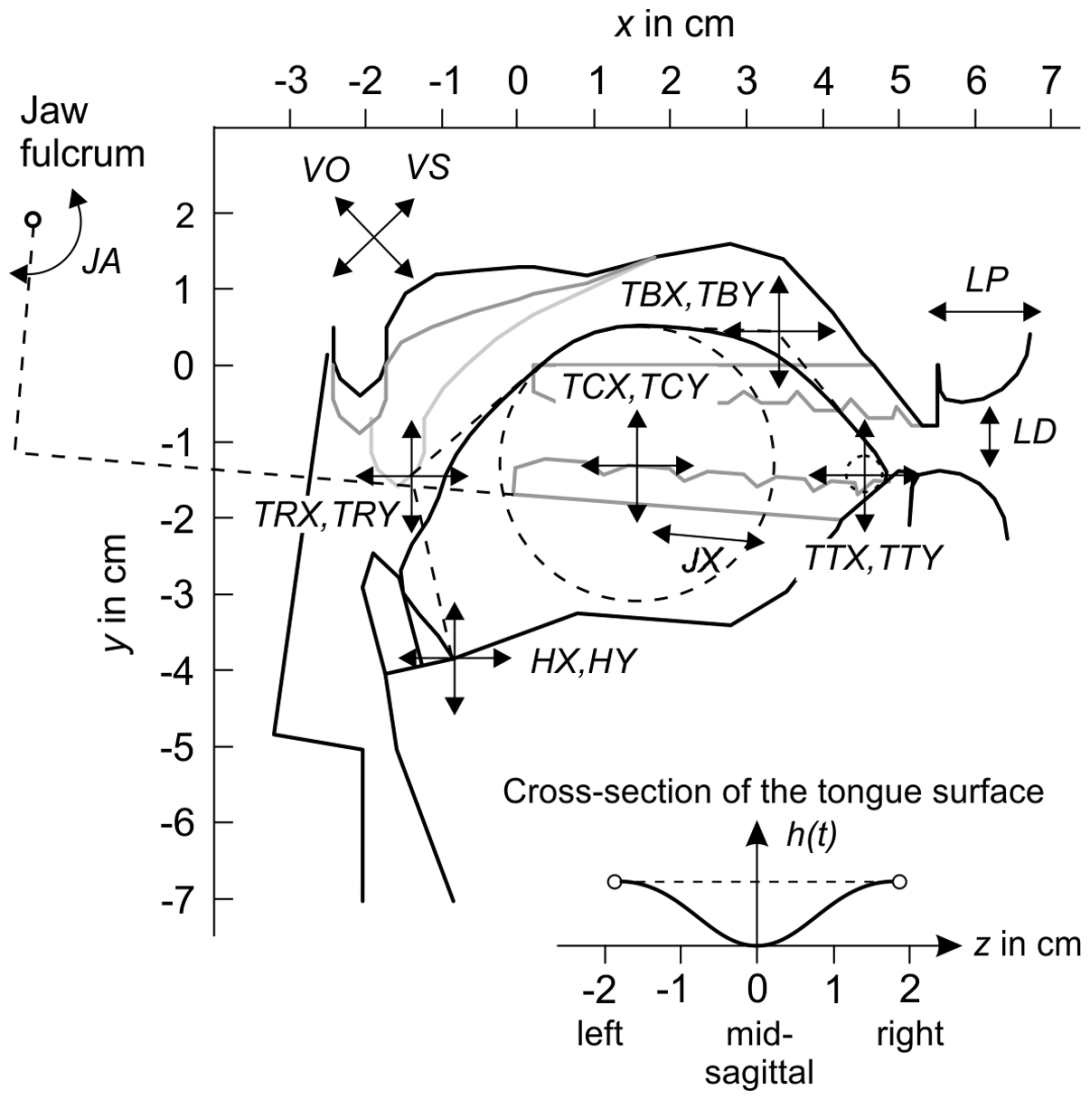
Areas of influence of the vocal tract parameters (refer to Table 2 for the abbreviations). The shape of the velum is controlled by *VO* and *VS*. The protrusion of the lips and the vertical distance between the upper and lower lip is specified by *LP* and *LD*, respectively. The horizontal and vertical position of the hyoid is specified by *HX* and *HY*, respectively. *JA* specifies the opening angle of the jaw and the *JX* its anterior-posterior translation. The tongue body and the tongue tip are modeled as circles with the center coordinates (*TCX, TCY*) and (*TTX, TTY*), respectively. The tongue root and the tongue blade are modeled with quadratic Bézier curves. The coordinates of the central control points of these curves are given by (*TRX, TRY*) and (*TBX, TBY*). The panel at the bottom illustrates the cross-section of the tongue at a position 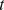 along the midsagittal tongue contour. *h(t)* defines the elevation of the tongue sides, which is specified by the parameters *TS1 ... TS4* at four equally-spaced positions along the tongue contour. Between these positions, *h(t)* is interpolated.

While the hard palate has a fixed position in the 3D coordinate system of the vocal tract, the jaw is assumed to execute rotational and translational movements controlled by the parameters *JA* and *JX*. *JA* defines the angle of rotation of an angle bracket around a transverse axis, as illustrated in Figure 7. The jaw is assumed to slide along the long lever of the angle bracket, and *JX* defines the displacement of this translational movement. The location of the fulcrum was estimated to model the dependency between the opening and the rotation of the jaw observed in the static MRI data. Note that it is more posterior than the actual anatomical mandibular joint. Thereby, a rotation around the fulcrum corresponds to a combined rotation and vertical translation of the jaw with respect to the real temporomandibular joint [37].

The velum shape was traditionally modeled by linear interpolation between two extreme positions: the highest possible position corresponding to a closed velo-pharyngeal port, and the lowest possible position corresponding to a maximally open port (e.g. [4], [12], [38]). However, the relation between the height of the velum and the acoustically-important velar opening area is difficult to determine with only one DOF for the velum. For example, our data showed considerable variations of the velum height for different vowels, all of which can be assumed to be produced with an essentially closed velo-pharyngeal port. A recent study actually found two independent DOF of the velum, both of which affect the velum shape and the velar opening area [39]. Therefore, we decided to model the function of the velum with two control parameters instead of one, namely the velum shape parameter *VS* and the velic opening parameter *VO*. *VS* defines the shape of the velum for a closed velo-pharyngeal port (*VO = 0*) by linear interpolation between a maximally raised position as in /s/, and a lowered position as in /a/. *VO* interpolates the final velum shape between the closed-port shape specified by *VS* and a maximally lowered shape as in /m/. The three reference shapes were modeled after the volumetric MR images of /s/ (*VS = 0* and *VO = 0*), /a/ (*VS = 1* and *VO = 0*), and /m/ (*VO = 1*), and are shown in Figure 7 as black, gray, and light gray contours, respectively. The velic area is assumed to be 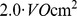 with negative areas being set to zero.

Two parameters, *HX* and *HY*, define the position of the hyoid and also determine the shape of the larynx, similar to Mermelsteins model [4]. *HY* defines the absolute vertical position of the hyoid and the larynx below. The actual shape of the larynx is linearly interpolated between two reference shapes for the narrowest (*HX = 0*) and the widest (*HX = 1*) larynx shapes observed in the volumetric MRI data. The shape of the lips is defined by the parameters *LP* and *LD*, which define the protrusion of the lip corners and the vertical distance between the upper and lower lip. From these parameters we derive all other important lip dimensions according to [40] and so construct the lip surfaces.

The 3D shape of the tongue is defined in terms of its midsagittal shape and the height of the tongue sides. The tongue body is represented by a circle with a fixed radius and a moving center defined by the absolute coordinates (*TCX,TCY*). The tongue tip is represented by a smaller second circle and the variable center coordinates (*TTX,TTY*). The root of the tongue is modeled with a quadratic Bézier curve defined by three control points. The first point is given by the hyoid position, the second point by the control parameters (*TRX,TRY*), and the third is the contact point of the tangent line to the tongue body circle that runs through (*TRX,TRY*). The tongue blade is also modeled with a quadratic Bézier curve where the second control point position is defined by the parameters (*TBX,TBY*), and the first and the third points are the contact points of the tangent lines to the tongue body circle and the tongue tip circle that run through (*TBX,TBY*). The two circles and the control polygons for the two splines are shown with dashed lined in Figure 7. The parameters *TS1, TS2, TS3*, and *TS4* define the height of the tongue sides with respect to the midsagittal contour at four equally-spaced positions between the hyoid and the tongue tip. This allows modeling of varying degrees of convex and concave cross-sections of the tongue surface along the midsagittal contour.

The acoustic properties of the vocal tract are essentially determined by the area function, i.e., the variation of the cross-sectional area as a function of the position between the glottis and the lips. The area function is determined by intersecting the vocal tract surfaces with planes perpendicular to the center line of the airway according to [36]. The calculation of the course of the center line is based on the position of the tongue body circle to dynamically adapt to the major shape variations of the vocal tract. Figure 6C shows as an example the area function for the vocal tract shape in Figure 6A. Because of the triangle mesh representation of the vocal tract surfaces, the precise cross-sectional areas in constricted regions of the vocal tract are sometimes difficult to control with the control parameters. However, for fricatives, the area of the constriction is a sensitive aerodynamic parameter for the flow resistance and the properties of the noise sources. To precisely control the cross-sectional area of constrictions, we introduced the parameters *MA1, MA2* and *MA3*, which define the minimal area in the regions upstream from the tongue tip, in the near vicinity of the tongue tip, and in the region of the incisors and lips, respectively. These parameters directly affect the area function, where they ensure the adjusted minimal areas in the corresponding regions.

### Modeling consonant-vowel coarticulation

To synthesize a sequence of phonemes with the model described above, the temporal variation of the vocal tract shape must be specified. In this study, we focus on the synthesis of consonant-vowel syllables as the most common and universal type of syllable in the world's languages [41]. Here, a CV syllable is modeled as a smooth unidirectional movement from an initial vocal tract shape appropriate for the consonant to a target shape for the vowel. The target shape for the vowel is assumed to be invariant, i.e., independent of the preceding consonant, as suggested in the undershoot model by Lindblom [42]. Hence, all potential variations of a vowel are assumed to be caused by vowel target undershoot. All other coarticulatory influences of consonants on vowels are currently considered as truly allophonic variations and would have to be modeled with different target shapes for different allophones of the vowels. In contrast to the vowel in the CV syllable, the vocal tract shape for the consonant is assumed to be context-sensitive [30], i.e., to vary depending on the context vowel. The context-sensitive consonant shape is derived as the weighted average of three reference shapes for that consonant in the context of the corner vowels /a/, /i/, and /u/. Before this approach is detailed further below, we discuss the adjustment of the vocal tract parameters for the vowels and the consonants in /a/, /i/, and /u/-context by means of the MRI-derived vocal tract contours and their acoustic optimization.

#### Reference vocal tract shapes for vowels

**Figure 8:**
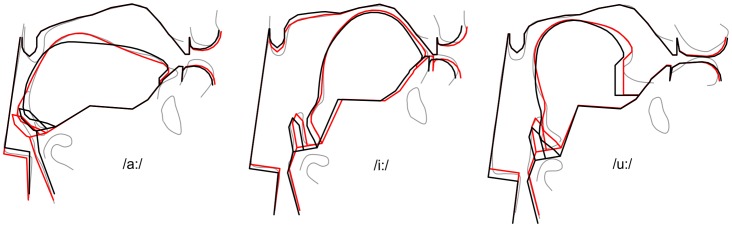
Midsagittal tracing of the vocal tract outline in the MRI data (gray), visually matched model contour (red), and acoustically optimized model shape (black) for the vowels /a:/, /i:/ and /u:/.

For each vowel analyzed in the volumetric MRI corpus, the vocal tract parameters were manually adjusted for the best possible visual match between the traced contours (including the tongue sides) and the vocal tract model contours. The flexibility of the vocal tract model allowed a fairly good match of the contours, as demonstrated in Figure 8 (red vs. gray contours).

Under ideal conditions, a close match between the vocal tract shapes of the reference speaker and the model would result in a close acoustic match. However, this was not always the case, because among other reasons, the vocal tract model is a non-perfect approximation of the real speaker's vocal tract, the accuracy of the traced contours in the MRI data was limited by the image resolution, and the artificially sustained vowels of the speaker in the MRI machine do not necessarily represent the ideal vowel targets. And in some regions of the vocal tract, even small deviations from the “correct” articulation can cause substantial acoustic changes [43]. Therefore, after the visual registration of the vocal tract shapes, the acoustic match between the natural and synthetic vowels was optimized. The goal was to minimize the acoustic deviations of *F1, F2* and *F3* between the model and the reference speaker by slight changes of the vocal tract shapes in terms of the control parameters. The acoustic error was defined as the root-mean-square of the relative error between the model-derived formants *F1, F2* and *F3*, and the speakers' formants *F1*′, *F2*′ and *F3*′ (according to Table 1):
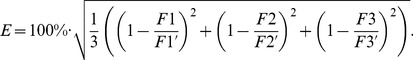
(1)


The model formants were determined from the volume-velocity transfer function of the vocal tract calculated in the frequency domain from the area function according to [14], [36]. To calculate the formants as accurately as possible, we considered acoustic energy losses due to sound radiation, soft walls, and viscous friction, as well as inner-length corrections of the vocal tract tube sections [44].

**Figure 9:**
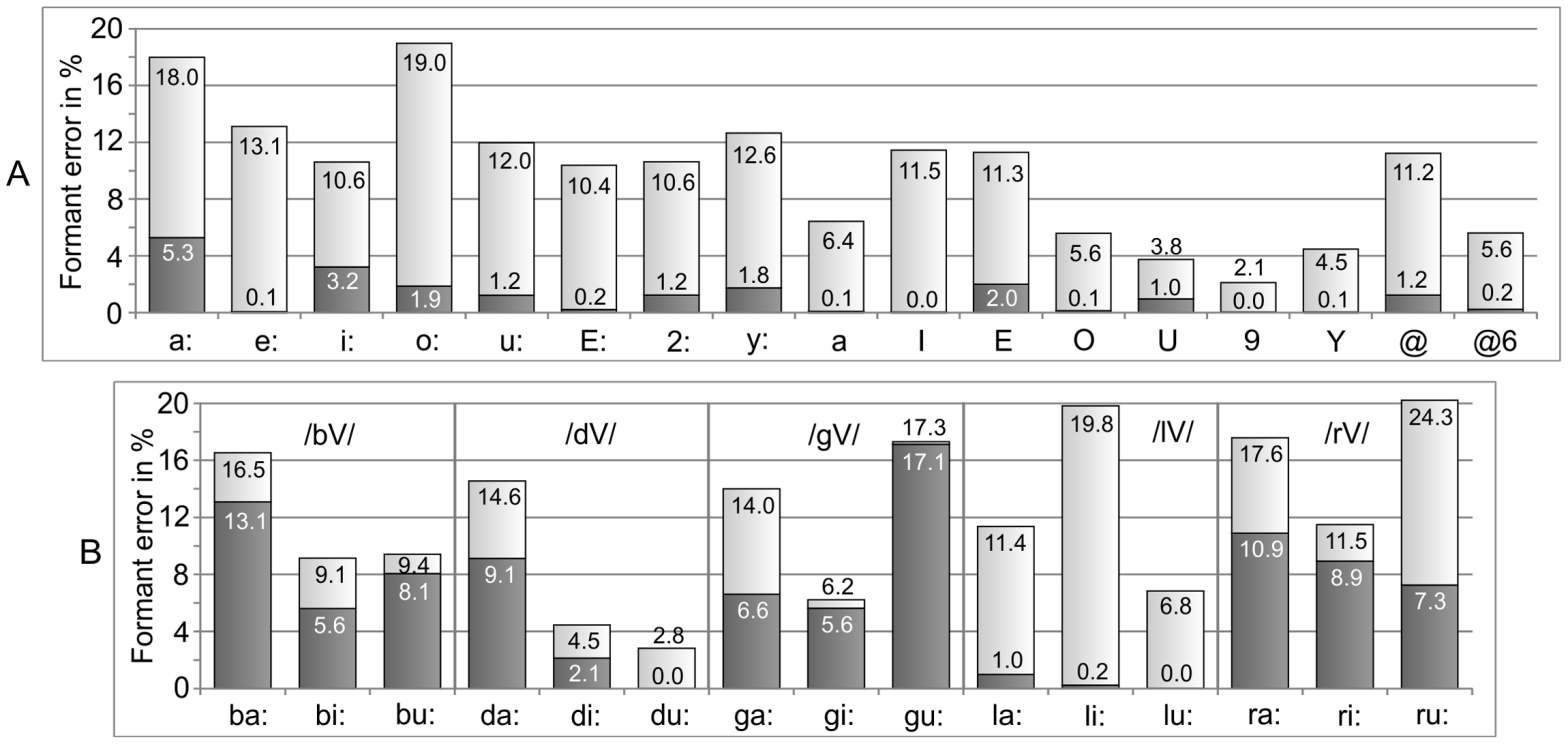
Acoustic errors of the modeled vowels and consonants before optimization (full bars) and after optimization (dark bars). A: Formant frequency deviations between modeled and recorded vowels. B: Onset formant frequency deviations between the modeled and measured consonants /b/, /d/, /g/, /l/ and /r/ in the context of the three corner vowels.

The optimization was implemented as a greedy algorithm. In each optimization step, the vocal tract parameter was identified for which a small positive or negative incremental change of the value resulted in the biggest reduction of the acoustic error *E*. After the incremental change had been applied to the parameter, the procedure was repeated for the resulting vocal tract shape until no reduction of *E* was possible anymore. The incremental changes of the parameters were defined such that the vocal tract outline displaced by no more than 0.5 mm per step. For the parameter *TTX*, for example, the possible increments were +0.5 mm and −0.5 mm. Two constraints were implemented: (1) The model contour of the vocal tract was not allowed to deviate more than a preset threshold from the initial MRI-fitted contour to keep the vocal tract shape geometrically similar to the MRI tracings. (2) The cross-sectional area of the vocal tract was not allowed to fall below a preset threshold to prevent unrealistically narrow constrictions that would cause excessive pressure drops or turbulence noise during the synthesis of the vowels [45]. For most vowels, the maximal contour displacement was set to 2 mm and the minimal area to 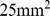. With these settings, the acoustic error reduced to below 5%, which is the just-discriminable change in the frequencies of the first and second formants [46]. However, for /a:/, /u:/, /2:/ and /y:/, the maximal allowed contour displacement had to be increased to 4 mm, and for /u:/ and /y:/, the area threshold had to be reduced to 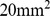 to achieve similar low errors. Figure 9A displays the acoustic errors for the vowels before (full bars) and after optimization (dark bars). On average, the error was 9.9% before and 1.2% after optimization. The black contours in Figure 8 illustrate the optimized vocal tract shapes for /a:/, /i/, and /u:/.

#### Reference vocal tract shapes for consonants

The normalized vocal tract tracings of the dynamic MRI corpus were used to model the context-sensitive targets for the consonants /b/, /d/, /g/, /l/, /r/, /m/, and /n/, each in the context of the vowels /a/, /i/, and /u/. Therefore, the vocal tract parameters were manually adjusted for a visual match with the MRI tracings analogously to the vowels. For /b/, /d/, /g/ and /l/, the articulatory data was directly available from the corpus. To model /r/, which was not recorded in the corpus, the tracings of /x/ were used, because /x/ and /r/ have the same place of articulation and differ mainly in the manner of articulation. In fact, the dorsal German /r/ may well be realized as a velar or uvular fricative in voiceless contexts such as the word “trat”. The vocal tract shape for /r/ in /i/-context, for which there was no data in the corpus, was manually modeled based on the contours of /g/ in /i/-context with a more retracted tongue. /m/ and /n/ were finally modeled based on /b/ and /d/ with a lowered velum as detailed further below.

**Figure 10:**
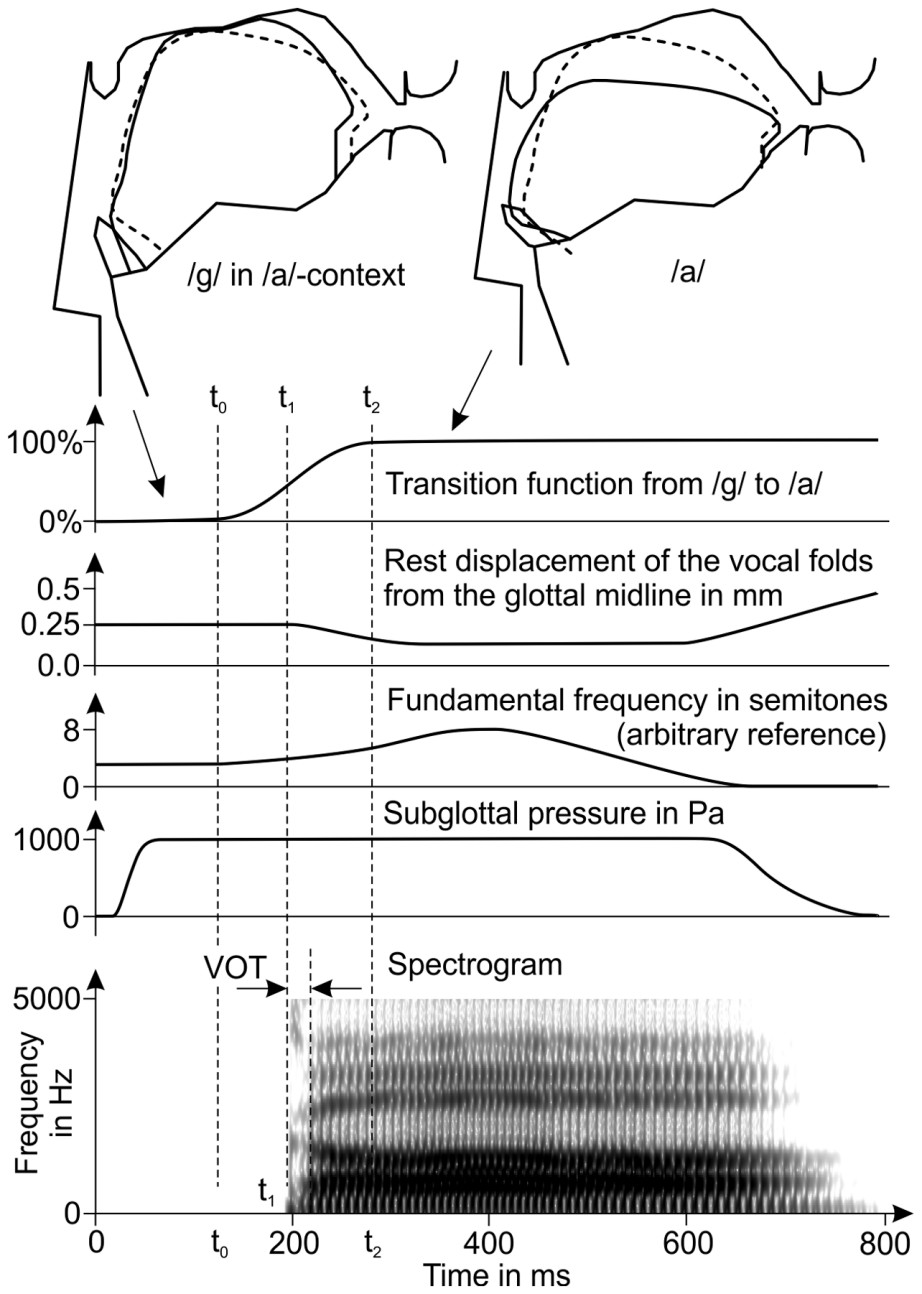
Vocal tract shapes and parameter time functions for the synthesis of the syllable /ga/. The solid vocal tract outlines show the vocal tract shapes for /g/ and /a/. The dashed contour shows the tongue shape at the time of closure release. 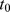, 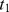, and 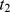 are the times of movement onset, closure release, and movement offset, respectively.

For each of the target shapes for /b/, /d/, and /g/, the vocal tract parameter(s) for the primary articulator were set to a *virtual target*, i.e., to a position that actually cannot be reached for the articulator. This allows for simulation of the high velocities of the primary articulators at the time of closure release in the framework of the target approximation model [47]. For example, the tongue tip target for /d/ was set to a position *above* the hard palate, and the lip distance for /b/ was set to a negative value. The virtual articulator positions were adjusted such that the release of the closure happened about in the middle of the transition from the context-sensitive consonant target to the corresponding context vowel target. For example, for the shape of /d/ in the context of /a/, the virtual target for the tongue tip was placed 10 mm above the hard palate, which coincides with the distance between the tongue tip and the palate in /a/. Figure 10 illustrates the effect of the virtual tongue body target of /g/ in the context of /a/. Here, the closure is released about half-way between the /g/-target and the /a/-target (dashed tongue contour). For the three context-sensitive targets of /l/, the tongue tip was set to positions where it just touched the palate, i.e., to non-virtual targets. The tongue sides were lowered to create lateral channels with a total cross-sectional area of 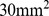, which falls well in the range of 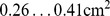 measured by Narayanan et al. [48]. The consonant /r/ was modeled as a voiced uvular fricative. For the three context-sensitive targets, the tongue body was set to a position where it just touched the velum. The control parameter *MA1* was used to adjust the constriction area to 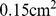, which is a typical minimal constriction area for voiced fricative consonants [49].

After the manual adjustment of the vocal tract contours, the consonant shapes were acoustically optimized similarly to the vowels. Here, the differences between the measured and synthesized formants at vowel onset (according to Table 1) were minimized, because these formant frequencies are important (although not the only) perceptual cues for the place of articulation [24], [50]. For /b/, /d/, /g/, and /r/, vowel onset was assumed to happen at the point along the linear transition from the context-sensitive consonant shape to the corresponding vowel shape where the constriction area increased to a value of 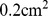. To our knowledge, due to the limitations of the current measurement technology, there are no precise data about the constriction area of plosives or fricatives at the time of voice onset. Therefore, the area of 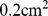 was estimated based on the data of voice onset times and rates of constriction area increase after plosives [45], [50]. For /l/, the formants were calculated directly from the consonant targets. For all consonant optimizations, the maximal allowed contour displacement was set to 4 mm instead of 2 mm as for the vowels, because of the greater uncertainties in the traced contours due to the low spatial and temporal resolution of the dynamic MRI data. The vocal tract parameters that defined the position of the primary articulator were not modified during the optimization.

The context-sensitive targets for /m/ and /n/ were modeled with the same optimized shapes as for /b/ and /d/ with the velic opening parameter *VO* set to 0.5 (corresponding to a velic opening area of 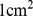). In some cases, the cross-sectional area between the tongue back and the lowered velum became unrealistically small. Therefore, if this area became smaller than 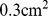, the tongue body position was adjusted as little as possible to establish a minimal area of 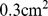.

**Figure 11:**
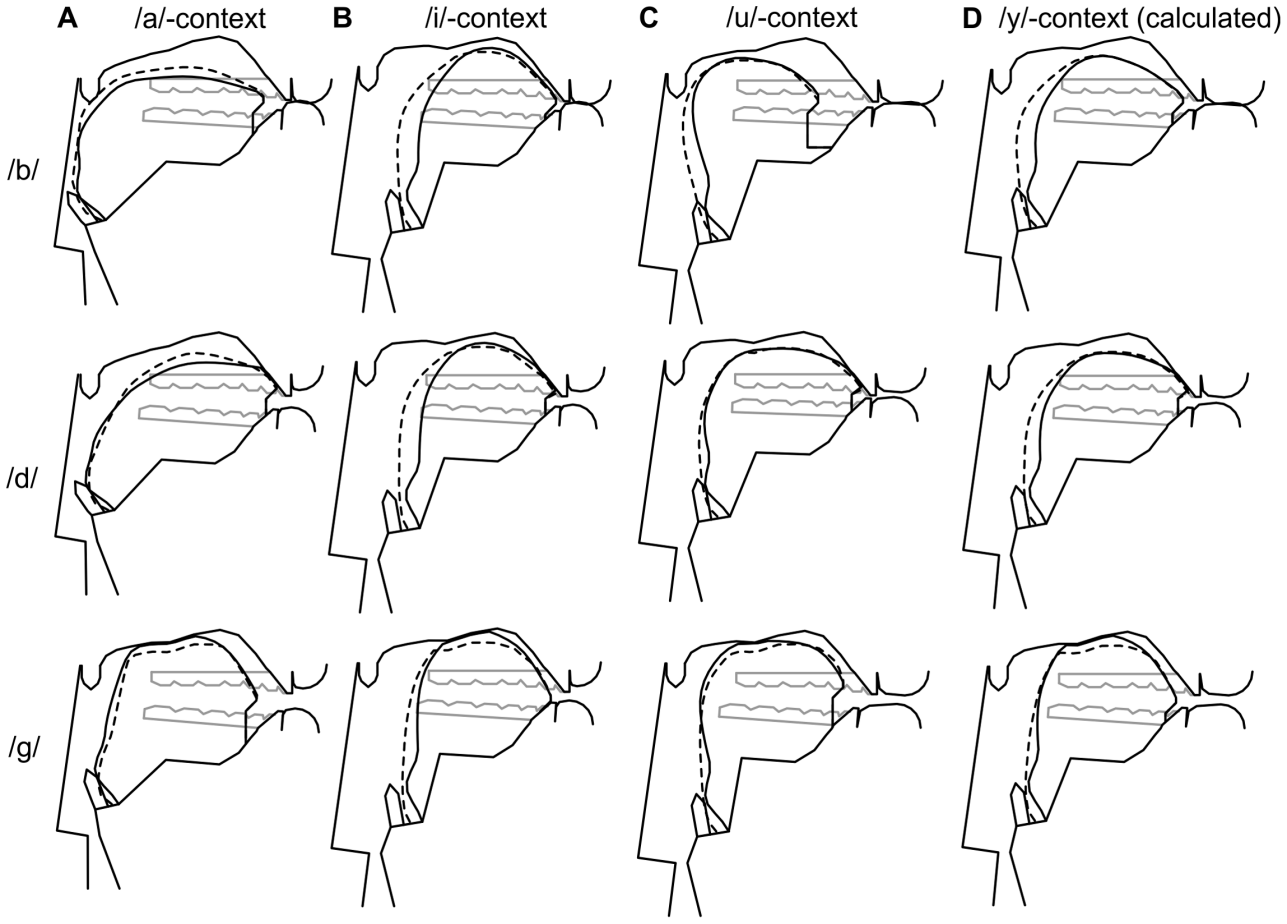
Modeled vocal tract shapes (after acoustic optimization) for /b/, /d/ and /g/ in the context of /a/, /i/, /u/, and /y/. A–C: Reference vocal tract shapes for /b/, /d/ and /g/ in the context of the corner vowels. The dashed lines indicate the contour of the tongue side. D: Calculated vocal tract targets for /b/, /d/ and /g/ in the context of the vowel /y/ according to the proposed coarticulation model.

Figure 9B shows the formant errors at voice onset before and after the acoustic optimization of the consonants. The *mean* acoustic errors of the consonants after optimization are given in the first row of Table 3. Figure 11A–C shows the optimized vocal tract targets for the consonants /b/, /d/, and /g/ in the context of the three corner vowels.

**Table 3:** Mean acoustic errors (%) of consonants.

Context vowels	/b/	/d/	/g/	/l/	/r/
/a/, /i/, /u/	8.9	3.7	9.8	0.4	9.0
All other vowels	9.7	9.1	8.6	7.7	15.7

The errors in % indicate the deviations between the measured and simulated formant frequencies (after optimization) at voice onset. The upper row shows the errors in the context of the corner vowels, for which the consonant targets were directly optimized. The lower row shows the error in the context of all other long and short vowels except /a, i, u/, for which the consonant target was derived using the proposed coarticulation model.

#### Modeling context-sensitive consonants

The basic assumption of our model is that the vowels /a/, /i/, and /u/ effectively represent the “corners” of the lingual vowel space, and that the measured consonant targets in the context of these vowels represent the corresponding extreme points of the consonants' lingual coarticulatory variation. The idea is to consider an arbitrary context vowel as a weighted average of the vowels /a/, /i/, and /u/, and derive a context-sensitive consonant target as the accordingly weighted average of the measured consonants in the contexts /a/, /i/, and /u/. However, vowels are not only distinguished by their lingual articulation, but also by the rounding of the lips. For example, /i/ and /y/ are characterized by roughly the same tongue position, but the lips are unrounded for /i/ and rounded for /y/. Therefore, the lip shape of a context-sensitive consonant is derived independently from the tongue shape based on the lip shape in the context vowel. Similar to the tongue shapes, the lip shapes of /a/, /i/ and /u/ can be roughly considered as extreme labial articulations for vowels in terms of lip protrusion and lip aperture (maximal aperture for /a/, maximal protrusion and minimal aperture for /u/, minimal protrusion for /i/).

As discussed before, the shape of the vocal tract is defined by the parameters given in Table 2. For the determination of a context-sensitive consonant target, these parameters are divided into two sets – one that represents the lingual articulation in terms of the vector 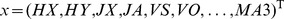, and one that represents the labial articulation in terms of the vector 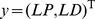 (hence, all vocal tract parameters except the lip parameters were considered to specify the “lingual” articulation). Let us first detail the calculation of the “lingual” parameters of a context-sensitive consonant. When the reference shapes for the vowels /a/, /i/, and /u/ are given by the vectors 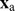, 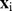, and 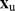, an arbitrary context vowel with the parameter vector 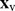 is expressed as a linear combination of these reference shapes:
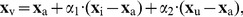
(2)or re-arranged



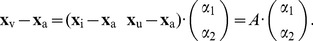
(3)The coefficients 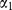 and 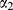 determine the position of 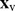 in the subspace defined by the three reference vowels. Because 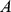 is likely to be singular, we use singular value decomposition to find a pseudo-inverse 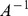 to determine 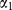 and 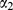:
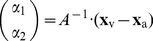
(4)


The coefficients are then used to obtain the context-sensitive consonant target as
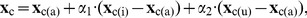
(5)where 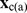, 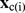, and 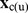 are the known parameter vectors for the consonant articulations in /a/, /i/, and /u/-context. Before 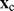 is actually calculated, the position 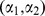 in the subspace is limited to the triangular region spanned by the reference vowels by enforcing the conditions 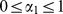, 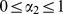, and 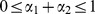.

In an analogous fashion to the lingual articulation, the labial articulation 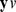 of an arbitrary context vowel is first expressed as the linear combination of the lip shapes for /a/, /i/, and /u/:
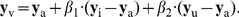
(6)


The system is solved for 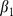 and 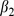, which are then limited analogously to 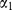 and 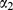. Finally, the lip shape for the context-sensitive consonant target is obtained as
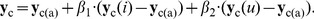
(7)


Figure 11D illustrates the method by means of the vocal tract targets for /b/, /d/ and /g/ in the context of the vowel /y/. When /y/ is mapped into the /a, i, u/-subspace, we get 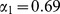, 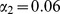, 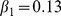 and 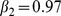. The lip shape lies slightly outside the triangle of the reference lip shapes, because 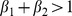. Therefore, 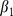 and 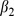 are reduced by equal amounts to 0.08 and 0.92 to satisfy the condition. The coefficients yield the linear combination 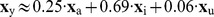 for the lingual articulation, and the combination 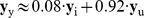 for the labial articulation. In other words, the lingual articulation of /y/ is most similar to /i/, and the labial articulation most similar to /u/. Putting 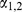 and 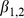 in Eqs. 5 and 7 results in the consonant targets in Figure 11D.

To check the effectiveness of the method for the *acoustic* simulation of the consonants, the target shapes for /b/, /d/, /g/, /l/ and /r/ were calculated in the context of all long and short German vowels *except* the corner vowels. For each of these CV-combinations, the vocal tract shape at the release of the consonantal constriction was calculated according to the previous section to obtain the formant frequencies at the vowel onset. The mean errors between the simulated and measured onset formant frequencies according to Eq. 1 are shown in the second row of Table 3. For /b/ and /d/, the error is roughly the same as for the consonants in the optimized reference contexts. For /d/, /l/ and /r/, the error increased by only 5.4, 7.3 and 6.7%. Hence, the proposed vocal tract shape interpolation allows a good simulation of the context-sensitive consonant acoustics across context vowels.

## Experiment

In order to assess the performance of the proposed coarticulation model, we tested the intelligibility of the modeled consonants in synthesized consonant-vowel syllables with a perceptual identification experiment.

### Stimuli creation

The stimuli consisted of CV-syllables for all combination of the modeled consonants /b/, /d/, /g/, /l/, /r/, /m/, /n/ and the long vowels /a:/, /e:/, /i:/, /o:/, /u:/, /E:/, /2:/, /y:/, i.e., a total of 56 items. The stimuli were created with the articulatory synthesizer VocalTractLab (www.vocaltractlab.de), where the proposed coarticulation model was implemented. The vocal apparatus in VocalTractLab was modeled as a branched acoustic tube system comprising the trachea, the glottis, and the pharyngeal, oral and nasal cavities [36]. The cross-sectional area function of the pharyngeal and oral cavities was determined by the vocal tract model presented above. The trachea was modeled as a uniform tube of 14 cm length, and the nasal cavity was modeled in terms of its area function according to the data of Dang et al. [51]. The glottis was represented by two tube sections, the geometry of which was determined by the glottal shape model proposed by Titze [52] and extended by Birkholz [36]. A detailed aero-acoustic simulation method generated the speech signal based on the acoustic tube system [14], [15], [36].

The stimuli were generated by specifying time-functions for the control parameters of the vocal tract model and the glottis model as illustrated in Figure 10. The parameters of the vocal tract model were initialized with the values for the context-sensitive consonant target and started to simultaneously approach their vowel target values at a time 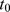. The transition was modeled as the step response of a critically damped sixth-order linear-system [53], which closely resembles the sigmoidal trajectory of natural goal-directed movements. The velocity of the vowel target approach was controlled by the time constant of the system. The time constant was individually estimated for each consonant by matching the formant transition durations of the synthetic syllables with naturally-spoken syllables. It was set to 7 ms for syllables with /l/, to 15 ms for syllables with /b/, /d/, /g/, and /r/, and to 25 ms for syllables with /m/ and /n/. A higher time constant causes a longer transition time and a slower velocity of the articulators than a lower time constant. Therefore, the simulated closure release of the nasals was generally slower than the release of the plosives, which is in agreement with measured data [45]. For the syllable /ga/ in Figure 10, the vowel target was reached at about the time 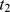, and the closure release happened at the time 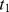. As discussed above, the release occurred about half-way between the (virtual) target for the consonant and the vowel target. The tongue contour at the time of the release is shown by dashed lines in the vocal tract images for /g/ and /a/.

The glottis model was controlled in terms of the degree of glottal abduction 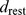 (pre-phonatory rest displacement of the inferior and superior edges of the vocal folds from the glottal midline at the level of the vocal processes), fundamental frequency 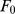, and subglottal pressure 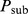. The time functions for 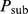 and 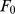 were adjusted identically for all stimuli. 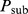 was quickly raised from 0 to 1000 Pa at the beginning of the syllable and smoothly lowered back to 0 Pa at the end. 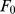 was reproduced from a spoken CV-syllable for a natural intonation. 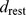 was initialized with a value appropriate for the consonant and started to approach the value for the vowel at about 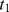. For the vowel, the 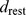 target was set to a value for modal phonation (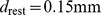). However, the initial 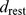 value was adjusted depending on the consonant. For /b/, /d/ and /g/, this parameter was used to produce different voice-onset times. According to our measurements, the mean VOT was 18 ms for /b/, 22 ms for /d/, and 38 ms for /g/. The rather short VOTs for /b/ and /d/ were roughly adjusted with a 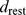 value appropriate for slightly-pressed (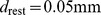) or modal (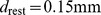) phonation. The longer VOTs for /g/ were generated with more abducted vocal folds during the consonant, which are typical for a slightly breathy type of phonation (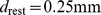). This is illustrated by the higher initial value for the glottal abduction in Figure 10. For /l/, /m/ and /n/, the degree of abduction was adjusted as for the vowel. The consonant /r/ was synthesized as voiced uvular fricative with slightly more abducted vocal folds to generate appropriate frication noise at the supraglottal constriction. After synthesis, the amplitude of the stimuli was normalized. All stimuli are contained in the supplemental materials Audio S1, Audio S2, Audio S3, Audio S4, Audio S5, Audio S6, and Audio S7.

### Subjects and method

The 56 stimuli were individually presented to 20 German listeners to identify the consonant and the vowel in each syllable. The participants, 9 men and 11 women, were native speakers of German (except one native English speaker, who has lived in Germany for more than 30 years) and 21–57 years old. Sixteen of them had a background in speech therapy or phonetics, and none of them reported any kind of hearing impairment. Each participant first listened to the eight isolated synthetic vowels to get used to the synthetic voice. Then, the stimuli were presented, in a different random order for each participant, in a quiet room using closed earphones. Each stimulus could be repeated once on request. The participants were asked to check one of the consonants “b”, “d”, “g”, “l”, “r”, “m”, “n”, and one of the long German vowels “a”, “e”, “i”, “o”, “u”, “ä”, “ö”, “ü” on a list after listening to each stimulus. They were asked to make their decisions spontaneously (but without an actual time limit) and to check the most similar phoneme in the case of uncertainty.

### Results and discussion

Recognition rates of the vowels and consonants in the synthetic syllables are shown in Table 4. With regard to the vowels, the recognition rates varied between 84.3% and 100.0%. However, statistically significant differences were only found between /i:/ and each of /a:/, /e:/, /o:/ and /E:/ by pairwise comparisons using Bonferroni corrected t-tests (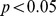). The overall recognition rate of 95.4% is close to that of natural vowels, e.g., in the study by Hillenbrand and Nearey [54]. They reported an average recognition rate of 96% for 12 English vowels in /hVd/ syllables. In the present study, /i:/ and /u:/ had the lowest recognition rate. The confusion matrix in Table 5 shows that 15.7% of the /i:/ vowels were falsely identified as /e:/, and 12.1% of /u:/ as /o:/. This indicates that the articulations of the corner vowels /i:/ and /u:/ were not extreme enough to differ sufficiently from their “neighbors” /e:/ and /o:/, respectively. As described before, the vowels were acoustically optimized with respect to the *mean* formant frequencies of 30 realizations in different contexts. However, the mean values do not apparently represent the asymptotic underlying targets for all vowels. In future work, considering the formant transitions towards the individual vowel samples could help to obtain more representative vowel targets.

**Table 4:** Recognition rates of the synthesized phonemes.

	Vowels									Consonants							
	a:	e:	i:	o:	u:	E:	2:	y:	all	b	d	g	l	r	m	n	all
Mean	100	100	84.3	100	87.1	100	97.1	94.3	95.4	73.1	71.9	83.1	100	81.3	67.5	100	82.4
S.D.	0.0	0.0	32.7	0.0	19.1	0.0	7.5	17.0	7.8	21.2	18.1	19.1	0.0	23.1	14.8	0.0	6.2
S.E.	0.0	0.0	7.3	0.0	4.3	0.0	1.7	3.8	1.7	4.7	4.0	4.3	0.0	5.2	3.3	0.0	1.4

Recognition rates, standard deviations (S.D.) and standard errors (S.E.) of the synthesized phonemes in percent (*N* = 20 subjects).

**Table 5:** Confusion matrix for vowels.

	Perceived vowel							
Vowel	a:	e:	i:	o:	u:	E:	2:	y:
a:	**100**	**.**	**.**	**.**	**.**	**.**	**.**	**.**
e:	.	**100**	**.**	**.**	**.**	**.**	**.**	**.**
i:	.	15.0	**84.3**	**.**	**.**	**.**	0.7	**.**
o:	.	.	.	**100**	**.**	**.**	**.**	**.**
u:	.	.	.	12.1	**87.1**	**.**	**.**	0.7
E:	.	.	.	.	.	**100**	**.**	**.**
2:	.	.	.	.	.	.	**97.1**	2.9
y:	.	.	.	.	.	.	5.7	**94.3**

Identification of the 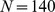 items per vowel in percent.

With regard to the consonants, the recognition rates varied between 67.5% and 100.0%. Pairwise comparisons of the recognition rates using Bonferroni corrected t-tests indicated that only the recognition rates of /n/ and /l/ (both of which were 100%) were significantly higher than those for all other consonants (/b/, /d/, /g/, /r/, and /m/) with 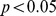. The overall recognition rate was 82.4%. When people listen to pseudowords produced by humans, they achieve a higher consonantal recognition rate, e.g., 99% for CV syllables as reported by Klatt [55], or 98% for the same seven consonants as in the present study in VCV syllables as reported by Broersma and Scharenborg [56]. This should be the goal or upper bound for all programs attempting to synthesize speech. To our knowledge, the present study is the first where the recognition of consonants generated with articulatory speech synthesis was systematically evaluated for a range of different context vowels. Hence, there is no directly comparable baseline with synthetic speech. Somewhat comparable to articulatory speech synthesis is formant synthesis, which is also a parametric speech synthesis technique. Formant synthesis was the dominating method for speech synthesis for several decades, until it was mostly displaced by concatenation systems in the 1990 s. During this time, the rules for generating intelligible phonemes were continuously improved and evaluated, and the consonantal recognition rate in nonsense CV syllables raised from about 75% in the first systems to about 95% in the best systems [55]. However, to achieve the 95% recognition rate, it was necessary to implement very detailed rules to describe the fine spectral details of the different consonants. In contrast, with the articulatory synthesis method in the present study, only a few simple control rules were necessary to achieve a consonantal recognition rate of already 82.4%.

Table 6 shows the confusion matrix of the consonants for a more detailed analysis. /l/ and /n/ were recognized 100% correct, indicating that the proposed coarticulation model can generally simulate the essential articulatory-acoustic variability of consonants. At the opposite end of the scale was /m/ with the lowest recognition rate. All of the falsely identified /m/ were recognized as /n/. This indicates that the feature “nasality” was well simulated by the model, but /m/ was not distinct enough from /n/. Apparently, the articulation of /m/ differs somewhat more from that of /b/ with a lowered velum as assumed in this study, Therefore, using actual articulatory measurements of the nasals to create their reference vocal tract shapes could improve their perceptual discrimination.

**Table 6:** Confusion matrix for consonants.

	Perceived consonant								Perceived consonant						
	b	d	g	l	r	m	n		b	d	g	l	r	m	n
ba:	**20**	.	.	.	.	.	.	da:	1	**8**	10	1	.	.	.
be:	**16**	.	3	1	.	.	.	de:	.	**13**	7	.	.	.	.
bi:	**13**	3	3	.	1	.	.	di:	.	**15**	5	.	.	.	.
bo:	**12**	.	1	.	7	.	.	do:	.	**20**	.	.	.	.	.
bu:	**14**	.	3	3	.	.	.	du:	.	**17**	3	.	.	.	.
bE:	**20**	.	.	.	.	.	.	dE:	1	**14**	4	.	1	.	.
b2:	**10**	1	8	.	1	.	.	d2:	.	**17**	3	.	.	.	.
by:	**12**	1	1	2	3	.	1	dy:	4	**11**	.	4	.	.	1
ga:	.	4	**16**	.	.	.	.	la:	.	.	.	**20**	.	.	.
ge:	.	5	**15**	.	.	.	.	le:	.	.	.	**20**	.	.	.
gi:	.	6	**14**	.	.	.	.	li:	.	.	.	**20**	.	.	.
go:	.	.	**20**	.	.	.	.	lo:	.	.	.	**20**	.	.	.
gu:	.	1	**19**	.	.	.	.	lu:	.	.	.	**20**	.	.	.
gE:	.	10	**10**	.	.	.	.	lE:	.	.	.	**20**	.	.	.
g2:	.	.	**20**	.	.	.	.	l2:	.	.	.	**20**	.	.	.
gy:	.	1	**19**	.	.	.	.	ly:	.	.	.	**20**	.	.	.
ra:	.	1	.	.	**19**	**.**	**.**	ma:	.	.	.	.	.	**19**	1
re:	.	7	1	1	**11**	**.**	**.**	me:	.	.	.	.	.	**1**	19
ri:	.	5	1	1	**13**	**.**	**.**	mi:	.	.	.	.	.	**7**	13
ro:	1	.	.	.	**19**	**.**	**.**	mo:	.	.	.	.	.	**20**	.
ru:	4	1	.	.	**15**	**.**	**.**	mu:	.	.	.	.	.	**20**	.
rE:	.	5	1	.	**14**	**.**	**.**	mE:	.	.	.	.	.	**8**	12
r2:	.	.	.	.	**20**	**.**	**.**	m2:	.	.	.	.	.	**20**	.
ry:	1	.	.	.	**19**	**.**	**.**	my:	.	.	.	.	.	**13**	7
na:	.	.	.	.	.	.	**20**								
ne:	.	.	.	.	.	.	**20**								
ni:	.	.	.	.	.	.	**20**								
no:	.	.	.	.	.	.	**20**								
nu:	.	.	.	.	.	.	**20**								
nE:	.	.	.	.	.	.	**20**								
n2:	.	.	.	.	.	.	**20**								
ny:	.	.	.	.	.	.	**20**								

Absolute number of responses for the consonants in the syllables for 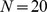 responses per syllable.

Of the plosives, /g/ had approximately a 10% (absolute) higher recognition rate than /b/ and /d/. However, the *acoustic* errors of the plosives in terms of formant onset frequencies were roughly equal according to Table 3. This indicates the importance of perceptual cues other than the formant transitions for the discrimination of the plosives. One is the VOT, which we roughly reproduced according to our measurements (Table 1). The better recognition of /g/ could in fact be brought forward by its clearly higher VOT compared to /b/ and /d/, because VOT was previously demonstrated to be highly effective in classifying place of articulation for plosives [50]. Another important perceptual cue of voiced plosives is the spectrum of the release burst [57]. In our simulations, the burst was automatically generated by our preliminary noise source model [15]. However, the simulation of noise sources in the time-varying vocal tract is a subject of extensive research in its own right and has not yet created a model that can be considered realistic and complete under all conditions. Therefore, a more realistic noise source model could substantially contribute to a better discrimination of the plosives.

The recognition rate of /r/, which was synthesized as a uvular voiced fricative, was 81.3%. The major confusions occurred in the context of the front vowels /i:/, /e:/ and /E:/. The reason could be that the context-sensitive vocal tract shape for /r/ in the context of /i/ had to be *estimated*, because it was not recorded with the real-time MRI corpus. Some participants reported after the experiment that they heard a /z/ instead of /r/ in the context of the front vowels, which indicates that the tongue position was too far anterior for the estimated reference shape and (or) that the noise source model generated noise sources that were too far downstream in the vocal tract or had inappropriate spectral properties.

## General Discussion

Currently, text-to-speech synthesis is completely dominated by concatenative and statistical parametric speech synthesis techniques. However, the increasing demands for highly expressive and flexible speech synthesis are recognized to be difficult to satisfy with these techniques. Therefore, articulatory speech synthesis is now becoming a serious alternative for speech synthesis again. In particular the increased availability of MR imaging makes it now possible to develop detailed quantitative models of the vocal tract and articulation. In this study we combined static MRI data, dynamic MRI data, and acoustic recordings of the same speaker to build a model of the vocal tract and coarticulation for the synthesis of consonant-vowel syllables. The key component of the model is the modeling of context-sensitive consonant targets based on a linear combination of measured consonant targets in the context of the corner vowels /a/, /i/, and /u/. The advantage of this phenomenological approach is that relatively little data is needed to model the coarticulatory variability of a consonant, and the data can be measured directly. Therefore, the application of the model to other phonemes, speakers, and languages is straightforward, for example using an analog available MRI corpus of a British English speaker [58]. In contrast to the consonants, vowels were modeled as invariant asymptotic targets according to the undershoot model [42].

To assess the performance of the model, it was used for the synthesis and perceptual evaluation of CV syllables for all combinations of seven consonants and eight vowels. For the vowels, the overall recognition rate of 95.4% was close to that of natural vowels. For the consonants, the overall recognition rate of 82.4% was still below the recognition rate of naturally spoken consonants (about 98% for the same set of consonants [56]). However, the detailed analysis of the recognition results clearly suggested the possible causes for confusions and the necessary steps to improve the synthesis of the consonants in future work. Currently, the most important limitation of the synthesizer is the noise source model, which generates the turbulence noise for fricatives and the bursts for plosives. The challenge for a noise source model for articulatory speech synthesis is to accurately predict the position, strength and spectral shape of noise sources based on the geometry and the aerodynamic conditions in the vocal tract. However, there is currently no noise source model that can be considered realistic and complete under all possible conditions. In this study, the plosives /b/, /d/, /g/, and the approximant /r/ involved noise sources for bursts and frication, respectively, all of which are known to be relevant perceptual cues for these phonemes [55], [57]. Therefore, future advances in the development of noise source models will likely increase the recognition rate for these consonants. Despite the current limitations, the achieved recognition performance seems to be already high enough for many text-to-speech applications where higher-level context could be expected to contribute to processes of word recognition and sentence comprehension. To demonstrate this, the supplemental materials Video S1 and Video S2 contain an example of a short German sentence synthesized with the proposed coarticulation model: “Lea und Doreen mögen Bananen. ” (“Lea and Doreen like bananas. ”, [le:aUndo:re:nm2:gNbana:n@n]). The fundamental frequency contour and the phone durations were reproduced from the same sentence spoken by the author of this study. The supplemental Image S1 shows the natural and synthesized sentences side-by-side in terms of oscillograms and spectrograms and illustrates the good agreement between the natural and synthetic formant transitions.

In this study, we only modeled CV syllables, but neither CVC syllables nor syllables with consonant clusters, which are necessary for unlimited speech synthesis. For these cases, the time structure model of the syllable [59] and models of CC coarticulation (e.g. [60]) offer initial guidance and will be the subject of future studies. Furthermore, it would be interesting to examine the intelligibility of the synthetic consonants when the degree of coarticulation is adapted in different steps between “full coarticulation” (as in this study) and context-independent articulation of consonants. The context-independent reference shape of a consonant could be obtained by averaging the three reference shapes in the context of the corner vowels to minimize contextual articulatory and acoustic variations. Much of the data analysis and model construction in this study was hand crafted, e.g., the tracing of the contours in the MR images, the manual adjustment of the vocal tract parameters for matching the model and MRI contours, and the determination of the formants in the acoustic recordings. In future work, at least some of these steps could be partly automated to save time when the model is adapted to new speakers and to increase reproducibility. A good candidate for automatization is contour tracing in the MR images, for which good results were achieved, e.g., by Bresch and Narayanan [61]. Finally, the noise source model should be improved to raise the overall recognition performance of the consonants.

## Supporting Information

Audio S1 **Synthetic stimuli with the consonant /b/ created for the perception experiment.**
(WAV)

Audio S2 **Synthetic stimuli with the consonant /d/ created for the perception experiment.**
(WAV)

Audio S3 **Synthetic stimuli with the consonant /g/ created for the perception experiment.**
(WAV)

Audio S4 **Synthetic stimuli with the consonant /l/ created for the perception experiment.**
(WAV)

Audio S5 **Synthetic stimuli with the consonant /r/ created for the perception experiment.**
(WAV)

Audio S6 **Synthetic stimuli with the consonant /m/ created for the perception experiment.**
(WAV)

Audio S7 **Synthetic stimuli with the consonant /n/ created for the perception experiment.**
(WAV)

Video S1 **The synthesized German sentence “Leo und Doreen mögen Bananen” with the vocal tract model rendered in 3D.** This sentence was reproduced from a naturally spoken sentence in terms of the fundamental frequency and the phone durations.
(AVI)

Video S2 **The same sentence as in Video S1 with the vocal tract model shown in the midsagittal plane.**
(AVI)

Image S1 **A comparison between the synthesized (top) and natural (bottom) spectrograms and oscillograms for the sentence in Video S1.**
(TIF)
