# Supplementary figures and images for: Modeling Consonant-Vowel Coarticulation for Articulatory Speech Synthesis
Source: PLoS One. 2013 Apr 16;8(4):e60603. doi: 10.1371/journal.pone.0060603 (PMC3628899; doi:10.1371/journal.pone.0060603)

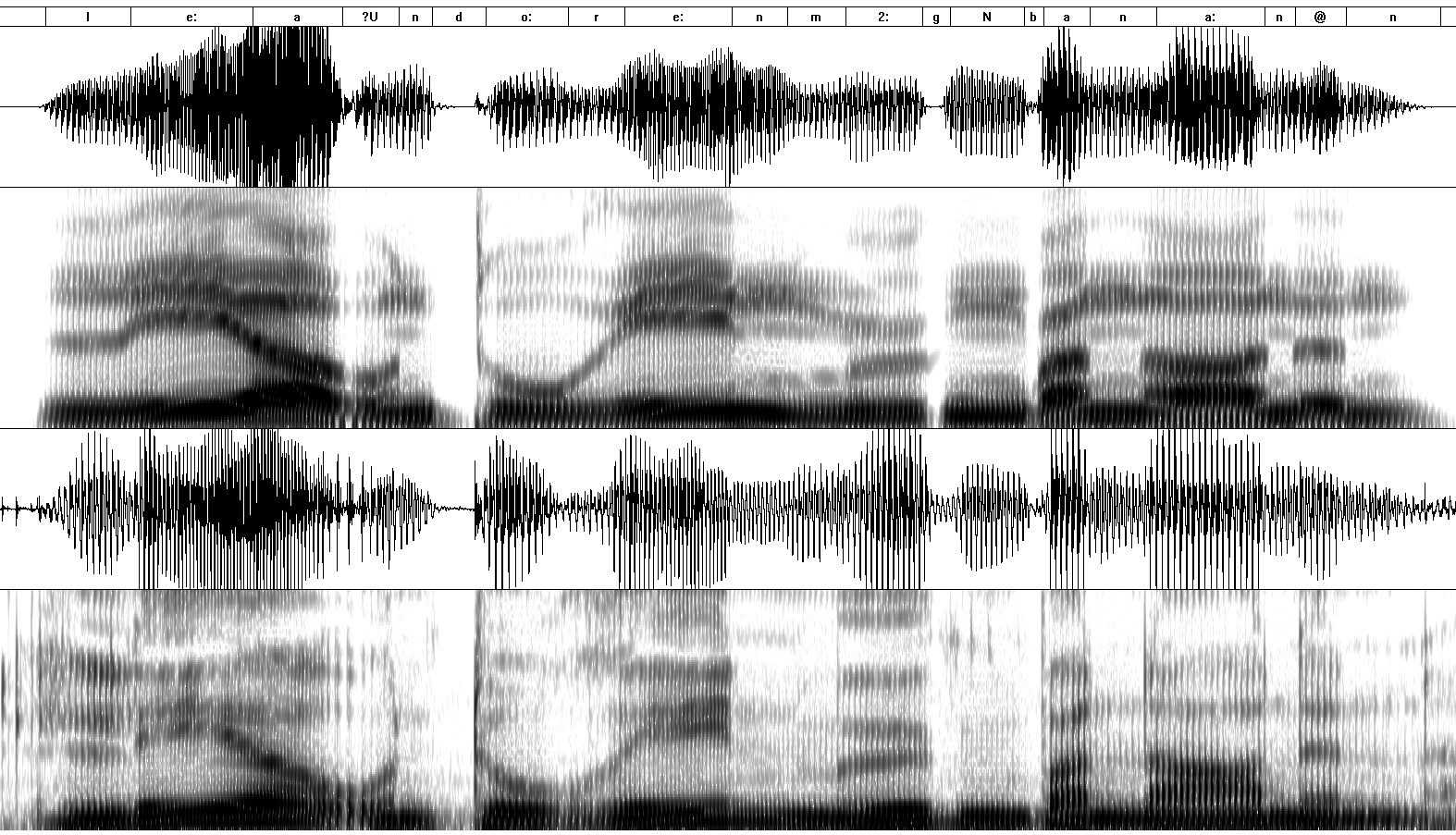

Supplement: Image S1 — A comparison between the synthesized (top) and natural (bottom) spectrograms and oscillograms for the sentence in Video S1. (TIF) [file pone.0060603.s010.tif]
